# Long-term cultivation shifts transcriptomic profile of HCT-116 cells

**DOI:** 10.17912/micropub.biology.002074

**Published:** 2026-04-22

**Authors:** Gudrun Marquardt, Theresa Wießner-Kroh, Stefan Rubner, Ioannis Papasotiriou

**Affiliations:** 1 Research Genetic Cancer Centre Central Europe GmbH, Weinbergweg 22, D-06120 Halle (Saale), Germany; 2 Research Genetic Cancer Centre International GmbH, Baarerstrasse 95, CH-6300 Zug, Switzerland

## Abstract

Human cancer cell lines are widely used in artificial culture systems to study cancer biology due to their immortalization, enabling extended culturing compared to primary cells. However, genomic instability can rapidly alter cellular behavior, raising questions about the transcriptome stability for controlled studies. We cultivated HCT-116 cells over 25 passages (~16 weeks), and observed a stable transcriptome up to passage 20, with only minimal differential gene expression. Above passage 20, genes corresponding to cell proliferation and cell death were upregulated suggesting a change in general cellular regulation and underlining defined cell culture time periods for stable and reproducible experimental conditions.

**Figure 1. Transcriptomic profile of 2D cultivated HCT-116 cells up to 25 passages.** **A.** Schematic workflow with arrows showing one passaging step. **B.** Volcano Plot of differentially expressed genes. Blue and red highlighted genes are significantly lower (p.adj. ≤ 0.05, log _2_ (FC) ≤ -1) or higher (p.adj. ≤ 0.05, log _2_ (FC) ≥ 1) expressed after 5 to 25 passages in 2D cultivation compared to cells after thawing. **C.** Heatmap of all significantly regulated genes showing the fold regulation after 5 to 25 passages in comparison to cells after thawing. Next to the fold change of each of the three biological replicates, the mean log _2_ (FC) together with statistical significance (stars for p-value < 0.05) are depicted. **D.** Heatmap of classical reference genes showing the fold regulation after 5 to 25 passages in comparison to cells after thawing. **E.** Selected clustered STRING networks of significantly differentially expressed genes after 25 passages of cultivation. The borders of the circles illustrate the log _2_ (FC) of the depicted genes. Clusters were subjected to functional enrichment and GO:BP terms with the highest enrichment score are shown. All data are shown as mean of three biological replicates.

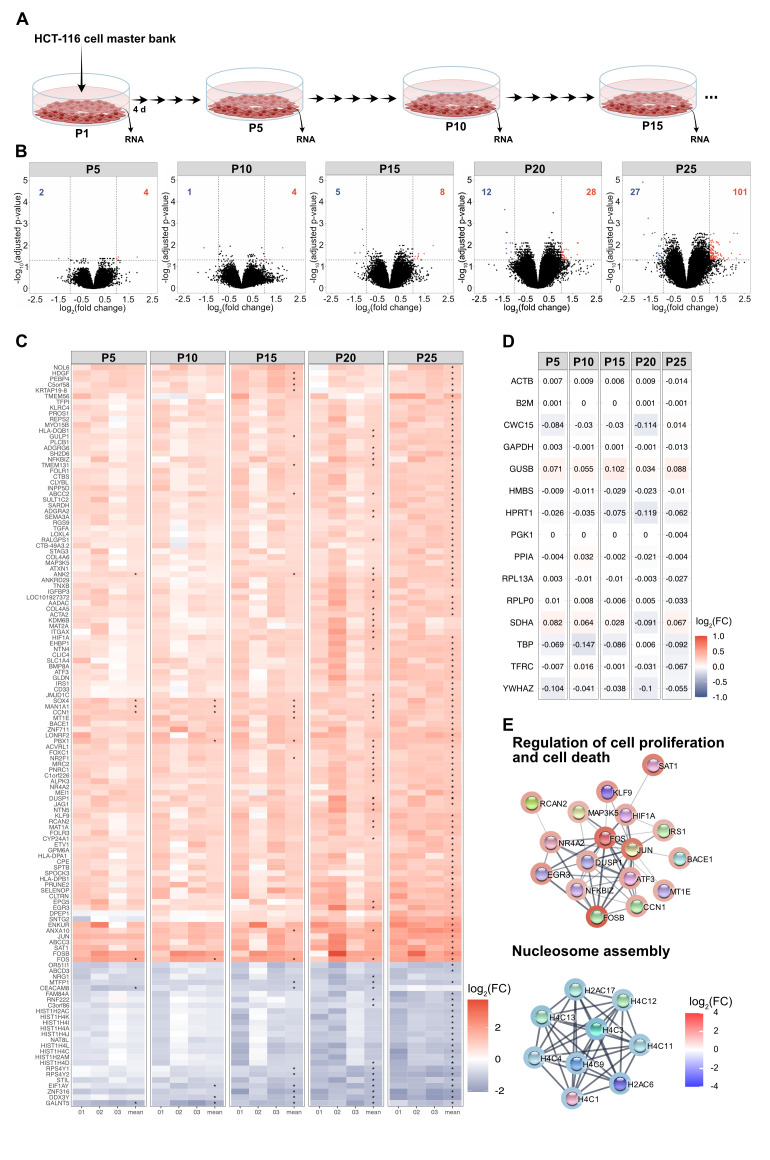



## Description

Cancer cells are widely employed as *in vitro* models for studying molecular and cellular mechanisms underlying tumor development, progression and developing therapeutic approaches. Unlike primary cells, established cancer cell lines possess the ability to proliferate indefinitely. This capacity for immortality is largely driven by mutations in key cell-cycle checkpoint regulators, among other genetic alterations (Albertson et al., 2003; Hanahan and Weinberg, 2000). These features enable prolonged cultivation and repeated experimentation, offering advantages over primary cells that have a very limited lifespan. However, one hallmark of cancer cells is genomic instability, which can lead to ongoing genetic and phenotypic changes during cell cultivation. Such instability may result in the selection of subpopulations with advantageous mutations, altering cellular behavior over time (Ben-David et al., 2018). This instability could also lead to gene copy number variations or even more to ploidy changes as shown for HeLa cell lines passaged over 50 passages (Y. Liu et al., 2019). Consequently, this raises an important question: within what time frame do established cancer cell lines remain sufficiently stable to provide reliable, robust and controlled conditions for cellular studies?

Several studies have examined how prolonged passaging affects cellular characteristics across different cell lines. In MIN-6 cells, for example, comparisons between early passages (17–19) and later passages (40–49) revealed nearly 1,000 differentially expressed genes, many of which are involved in insulin secretion, proliferation, and cell adhesion (O’Driscoll et al., 2006). In LNCaP cells, differences between passage 25 and passage 60 indicate alterations in the PI3K/Akt signaling pathway—a central regulator of cell growth, metabolism, apoptosis, and migration—ultimately leading to changes in androgen receptor protein levels (Lin et al., 2003). Metabolic profiles are also affected during passaging in HT29 and RAW 264.7 cells (Abdul-Hamid et al., 2019; Cao et al., 2021). In addition, cell lines such as MCF‑7, D1, ACHN, Renca and PC12 exhibit passage‑dependent shifts in gene expression and cellular behavior (Hamadneh et al., 2018; Kinarivala et al., 2017; Kwist et al., 2016; S. Liu et al., 2025). Collectively, these findings demonstrate that essential pathways can drift over time in a cell‑type‑specific manner, highlighting the importance of evaluating transcriptomic stability for any cell line under study.

HCT-116 is a widely utilized model cell line for studying colon cancer, frequently cited in scientific literature. However, some publications do not specify the passage number of HCT-116 cells as well as the time frame in which cellular experiments were performed, which can lead to inconsistencies in experimental results. To ensure stable conditions in our studies, we analyzed the transcriptome-wide gene expression of HCT-116 cells during cell cultivation over approximately 16 weeks passaging cells every 4 to 5 days. This evaluation aims to determine the passage number and time frame at which the cell line remains stable for consistent experimental outcomes. Therefore, RNA was isolated for every 5 passages until passage 25 and mRNA was analyzed by microarray ( Figure 1 A). During this time frame, a population doubling number of approximately 130 was reached, calculated from a doubling time of 20.0&nbsp;±&nbsp;0.8 hours determined over a period of 4 days.

Interestingly, differential gene expression was observed primarily for single genes during the first passages with up to 5 significantly downregulated and 8 significantly upregulated genes for passages 5 to 15 ( Figure 1 B). In subsequent passages, there was a notable increase in the number of differentially regulated genes ( Figure 1 B). Furthermore, the fold change of differentially expressed genes (DEGs) continued to rise over time, reaching its maximum at passage 25 ( Figure 1 C). Notably, the DEGs remained consistent across all passages, underscoring the increasing effect on these DEGs throughout the progression of passaging. Classical reference genes such as β-actin (ACTB), glyceraldehyde-3-phosphate dehydrogenase (GAPDH), tyrosine 3-monooxygenase/tryptophan 5-monooxygenase activation protein, ζ polypeptide (YWHAZ), CWC15 spliceosome-associated protein (CWC15), and TATA-binding protein (TBP) were not regulated within this time frame ( Figure 1 D). A significant number of the deregulated genes is involved in the regulation of cell proliferation and cell death (upregulated) as well as nucleosome assembly (downregulated) ( Figure 1 E). Notably, two members of the Fos gene family – FOS and FOSB – were upregulated. Both genes are well‑characterized proto‑oncogenes involved in regulating a broad spectrum of downstream targets. Among others, they interact with and modulate the AP‑1 transcription factor complex, thereby influencing processes such as cell proliferation, differentiation, and stress responses (Cordier and Creytens, 2023). Another gene upregulated in higher passages within this regulatory context was JUN, which is known to form heterodimers with FOS proteins to constitute the functional AP‑1 complex (Jafri et al., 2025). This upregulation can potentially enhance the genomic drift of the HCT-116 cell line. About one third of the downregulated genes were genes encoding for histone subunit variants. Changes in histone assemblies could affect genome stability as they have important functions in DNA repair mechanisms (Phillips and Gunjan, 2022; Vijayalakshmi et. al., 2025).

In summary, this observation indicates an overall stable HCT-116 gene expression during the first 10 weeks after starting the cell culture corresponding to passage 15. However, changes in gene expression became apparent with cultivation duration. Especially the change of cell proliferation and cell death related genes could change cellular behavior and responses to therapies. Our study highlights that low passage numbers or short cultivation times are essential for maintaining well‑controlled experimental conditions.

## Methods

**Cell culture**

The human colon carcinoma cell line HCT-116 (ECACC 91091005) was cultivated as recommended in McCoy’s 5A supplemented with 10&nbsp;% fetal bovine serum (FBS) and 1&nbsp;% Penicillin/Streptomycin. HCT-116 cells were seeded in TC-treated culture flasks (Corning) and grown for the indicated time until reaching 70 to 90&nbsp;% confluency per passage. Cell cultivation and all incubation steps were performed at 37&nbsp;°C, 5&nbsp;% CO _2_ and 95&nbsp;% humidity. Unless otherwise stated, cell culture medium and consumables were used from Life Technologies. HCT-116 cells were regularly tested negative for mycoplasma contamination by PCR (Takara Bio; Transgen Biotech) and authentication was confirmed by STR analysis (Eurofins).

**RNA isolation and gene expression analysis via microarray**

RNA was isolated from cultivated cells at 70&nbsp;% confluency. Cells were washed two times with DPBS and RNA was isolated via RNeasy Mini kit (Qiagen) with an additional on-column DNase I (Qiagen) digest according to the manufacturer’s instructions.

The transcriptome-wide analysis was conducted using a one-color microarray as described by Agilent. Briefly, 200&nbsp;ng of total RNA were reversely transcribed to cDNA, further transcribed to cRNA and labelled with Cyanin&nbsp;3-CTP via the Low Input Quick Amp Labelling Kit (Agilent, 5190-2305). Labeled cRNA was purified by using the RNeasy Mini kit (Qiagen) without on-column DNase digest. 600&nbsp;ng purified cRNA was fragmented according to the Gene Expression Hybridization Kit (Agilent, 5188-5242) and transferred to Gasket slides (Agilent, G2534-60014). The microarray chip SurePrint G3 human gene expression 8x60k v3 (Agilent, G4851C) was added to Gasket slide to form the hybridization sandwich which was fixed via the hybridization chamber (Agient, G2534A) and subsequently incubated at 65&nbsp;°C for 17&nbsp;h while rotation. Unbound cRNA was removed using the Gene Expression Wash Buffer Kit (Agilent, 5188-5327) followed by the immediate scan of the prepared microarray chip by microarray scanner InnoScan910 (Innopsys). InnoScan910 controlling and raw data extraction was performed via the Mapix software (Innopsys).

In total, three biological replicates, each replicate grown independently from an individual thawed cell culture vial, were analyzed.

**Microarray data analysis**

Microarray data were analyzed using R (Team, 2014) and Bioconductor (Gentleman et al., 2004; Huber et al., 2015) with the following associated packages: biomaRt (Durinck et al., 2005, 2009), dendextend (Galili, 2015), dplyr (Wickham et al., 2023), fgsea (Korotkevich et al., 2016), ggplot2 (Wickham, 2016), ggsci (Xiao, 2023), ggthemes (Arnold, 2021), ggVennDiagram (Gao, 2022), limma (Ritchie et al., 2015), msigdb (Bhuva et al., 2022), pheatmap (Kolde, 2019), tidyverse (Wickham et al., 2019). Raw intensities were log _2_ -transformed, background corrected using the normexp method and an offset of 50, and quantile normalized. Microarray probes were filtered for protein-coding genes and for genes expressed in at least one sample group. Differentially expressed genes were identified by fitting linear models (limma package) and using moderated t-statistics and Benjamini and Hochberg's p-value adjustment (Benjamini and Hochberg, 1995).

Based on significantly differentially expressed genes, networks were generated with Cytoscape (Shannon et al., 2003) based on the STRING database of protein interactions (Szklarczyk et al., 2023; von Mering et al., 2003, 2005). The STRING network was clustered using the STRING app included Markov Cluster algorithm (MCL) (Van Dongen, 2008) with the default inflation value of 4. These clusters were functionally annotated based on the GO:BP database (Ashburner et al., 2000; The Gene Ontology Consortium et al., 2023).
